# Correlation between antibiotic consumption and the incidence of healthcare facility-onset *Clostridioides difficile* infection: a retrospective chart review and analysis

**DOI:** 10.1186/s13756-021-00986-9

**Published:** 2021-08-06

**Authors:** Ji Hyun Yun, Ga Eun Park, Hyun Kyun Ki

**Affiliations:** Division of Infectious Diseases, Department of Internal Medicine, School of Medicine, Konkuk University, Konkuk University Medical Center, 120-1 Neungdong-ro, Gwangjin-gu, Seoul, 05029 Republic of Korea

**Keywords:** Antibiotics, Consumption, Correlation, Healthcare facility-onset *Clostridium difficile* infection, Incidence

## Abstract

**Background:**

Healthcare facility-onset *Clostridioides difficile* infection is the leading cause of antibiotic-associated diarrhea, and is associated with morbidity and mortality. The use of antibiotics is an important risk factor for healthcare facility-onset *C. difficile* infection. We evaluated the correlation between the incidence of healthcare facility-onset *C. difficile* infection and antibiotic consumption, according to antibiotic class.

**Methods:**

Patients with healthcare facility-onset *C. difficile* infection from January 2017 to December 2018 at Konkuk University Medical Center (a tertiary medical center) were included. We evaluated changes in the incidence of healthcare facility-onset *C. difficile* infection and antibiotic consumption. The correlation between the incidence of healthcare facility-onset *C. difficile* infection and antibiotic consumption was evaluated two ways: without a time interval and with 1-month interval matching.

**Results:**

A total of 446 episodes of healthcare facility-onset *C. difficile* infection occurred during the study period. The incidence of healthcare facility-onset *C. difficile* infection was 9.3 episodes per 10,000 patient-days, and increased significantly. We observed an increase in the consumption of β-lactam/β-lactamase inhibitors, and a decrease in the consumption of other classes of antibiotics, with a significant decrease in the consumption of fluoroquinolones, glycopeptides, and clindamycin (*P* = 0.01, *P* < 0.001, and *P* = 0.001, respectively). The consumption of β-lactam/β-lactamase inhibitors was independently correlated with the incidence of healthcare facility-onset *C. difficile* infection in the analysis without a time interval. When the analysis was conducted with 1-month interval matching, glycopeptide consumption was independently associated with the incidence of healthcare facility-onset *C. difficile* infection.

**Conclusions:**

Despite the reduction in fluoroquinolone and clindamycin consumption, the incidence of healthcare facility-onset *C. difficile* infection increased during the study period, and was correlated with increased consumption of β-lactam/β-lactamase inhibitors. Reduced consumption of specific antibiotics may be insufficient to reduce the incidence of healthcare facility-onset *C. difficile* infection.

**Supplementary Information:**

The online version contains supplementary material available at 10.1186/s13756-021-00986-9.

## Background

*Clostridioides difficile* infection (CDI) is a major cause of diarrhea in hospitalized patients [1]. The clinical manifestations are diverse, from asymptomatic carriage to life-threatening conditions, such as toxic megacolon, shock, and death [2]. A worldwide increase in the incidence of CDI has been reported, and is associated with the wide use of broad-spectrum antibiotics, the emergence of hypervirulent strains, and the use of more sensitive diagnostic tools, such as nucleic acid amplification tests (NAATs) [3–7]. There have been many attempts to reduce the incidence of healthcare facility-onset (HO)-CDI, including antibiotic stewardship programs and infection control measures [8–15]. Recently, the United States Centers for Disease Control and Prevention reported a decrease in the incidence of CDI, predominantly in HO-CDI [16].

The consumption of antibiotics, such as ampicillin, cephalosporins, clindamycin, and fluoroquinolones, is an important risk factor for CDI, and is associated with the incidence of HO-CDI [1, 6, 17, 18]. Furthermore, a reduction in antibiotic consumption, due to antibiotic stewardship programs, resulted in a decrease in the incidence of HO-CDI [8–10, 12, 14]. These studies were conducted in Western countries, such as the United States and Europe. However, because of the differences in the major strains and their antibiotic susceptibility, the effect of antibiotic stewardship programs on the incidence of HO-CDI may differ depending on the nation [19].

In this study, we evaluated changes in the incidence of HO-CDI and the consumption of commonly used antibiotics, in terms of defined daily dose (DDD) and days of therapy (DOT). The correlation between the incidence of HO-CDI and antibiotic consumption was evaluated two ways, according to the class of antibiotics: without a time interval and with 1-month interval matching.

## Methods

### Study population and design

We retrospectively reviewed the medical records of patients with HO-CDI at Konkuk University Medical Center, an 800-bed tertiary hospital, from January 2017 to December 2018. The incidence of HO-CDI was evaluated monthly. It was measured in CDI episodes per 10,000 patient-days, and its trend was evaluated during the study period. Antibiotics, including intravenous and oral antibiotics, commonly used in hospitalized patients were classified as β-lactam/β-lactamase inhibitors (BLBLIs), third-generation cephalosporins, fourth-generation cephalosporins, fluoroquinolones, carbapenems, tigecycline, and clindamycin. The following antibiotics were included in each class: BLBLIs: ampicillin/sulbactam, amoxicillin/clavulanate, and piperacillin/tazobactam; third-generation cephalosporins: cefotaxime, ceftriaxone, ceftazidime, cefpodoxime, cefixime, cefditoren, and cefdinir; fourth-generation cephalosporins: cefepime; fluoroquinolones: ciprofloxacin, levofloxacin, and moxifloxacin; carbapenems: and ertapenem, meropenem, and imipenem. Antibiotic consumption during the study period was evaluated monthly and measured in DDD per 1000 patient-days and DOT per 1000 patient-days. The incidence of HO-CDI and the consumption of antibiotics, which were measured monthly, were paired without time intervals and with 1-month intervals (e.g., antibiotic consumption in January 2016 was matched with the incidence of HO-CDI in February 2016). The correlation between the incidence of HO-CDI and the consumption of each class of antibiotics was evaluated two ways, to determine the immediate and delayed effects of antibiotic consumption on the incidence of HO-CDI. The study was approved by the Institutional Review Board of Konkuk University Medical Center. The requirement for written informed consent was waived owing to the retrospective nature of the study.

### Diagnosis of HO-CDI

We diagnosed HO-CDI according to the United States Infectious Disease Society guidelines [13] when patients had associated symptoms (unformed stools ≥ three times/day) and a positive test for *C. difficile* (a two-step glutamate dehydrogenase assay and a NAAT or a NAAT alone) after 3 days of hospitalization. Patient stool samples were tested for the presence of the *tcdB* gene (which encodes toxin B) using the Xpert^®^ *C. difficile* assay (Cepheid; Sunnyvale, CA, USA) according to the manufacturer’s instructions [20].

### Antibiotic stewardship program

The antibiotic stewardship program began in our hospital in March 2005. Initially, the program included broad-spectrum antibiotics, such as fourth-generation cephalosporins, piperacillin/tazobactam, carbapenems, tigecycline, glycopeptides, linezolid, and aminoglycosides. Thereafter, third-generation cephalosporins, fluoroquinolones, ampicillin/sulbactam, amoxicillin/clavulanate, and metronidazole were additionally included beginning in 2007. In 2009, the prophylactic use of third-generation cephalosporins and aminoglycosides was restricted. Professors in the Department of Infectious Diseases reviewed antibiotics ordered by physicians and determined their daily use. Without agreement from the reviewer, physicians could not order antibiotics for more than one day. Antibiotic consumption was monitored by hospital pharmacists. Data on antibiotic consumption were obtained from the centralized hospital database.

### Statistical analyses

Trends in the incidence of HO-CDI were evaluated using Poisson regression analysis. Changes in antibiotic consumption during the study period were evaluated using linear regression analysis. Correlations between the incidence of HO-CDI and antibiotic consumption were evaluated using Spearman’s correlation analysis and linear regression analysis. Variables with *P* < 0.1 in the linear regression analysis were included for further evaluation. All statistical analyses were conducted using SPSS version 21.0 (SPSS Inc., Chicago, IL, USA). *P* < 0.05 was considered statistically significant.

## Results

### Incidence of HO-CDI

During the study period, a total of 586 CDI episodes occurred. We excluded 140 episodes because they occurred within 3 days after admission, meaning they were community acquired. The remaining 446 CDI episodes were included in this study.

**Fig. 1:**
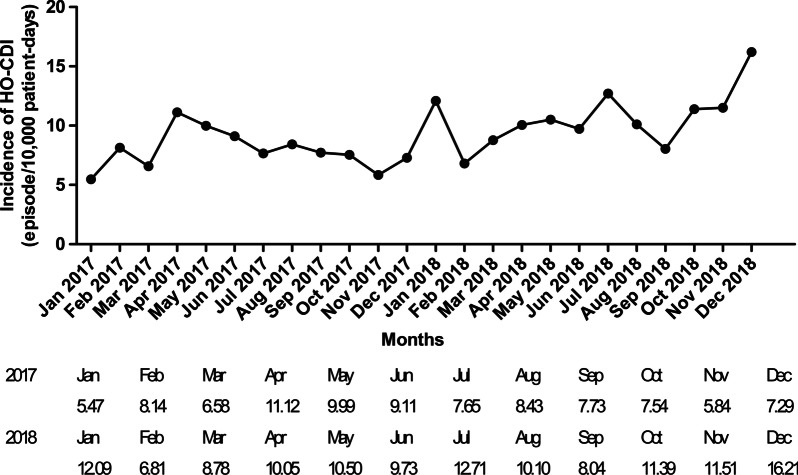
The incidence of HO-CDI during the study period. The incidence of HO-CDI was measured monthly. During the study period (from January 2017 to December 2018), the incidence of HO-CDI significantly increased (*P* = 0.001). *HO-CDI* healthcare facility-onset *C. difficile* infection

The mean incidence of HO-CDI was 9.3 episodes per 10,000 patient-days (95% confidence interval: 8.3–10.3 episodes per 10,000 patient-days) during the study period. The incidence of HO-CDI significantly increased 1.8 times from January 2017 to December 2018 (*P* = 0.001; Fig. 1). When the outlying data points (January 2017 and December 2018) were excluded, the incidence of HO-CDI showed an increasing trend, without statistical significance (*P* = 0.054).

### Antibiotic consumption

**Fig. 2:**
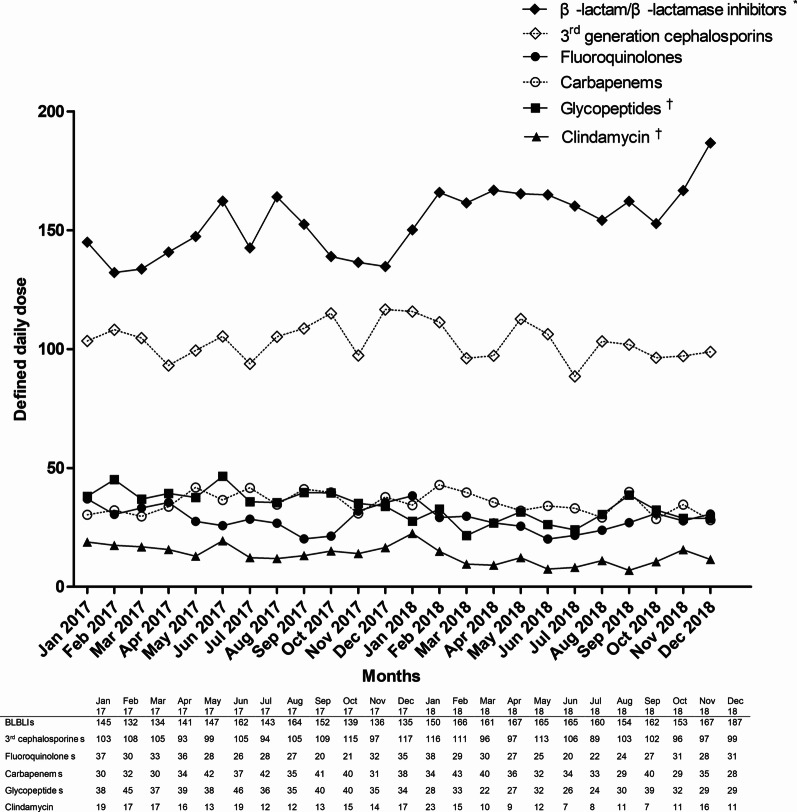
The trend of antibiotic consumption with defined daily doses during the study period. The consumption of β-lactam/β-lactamase inhibitors significantly increased, while the consumption of glycopeptides and clindamycin significantly decreased. *Indicates the antibiotics whose use increased significantly and † indicates the antibiotics whose use decreased significantly. *BLBLIs* β-lactam/β-lactamase inhibitors, *3rd cephalosporins* third-generation cephalosporins

**Fig. 3:**
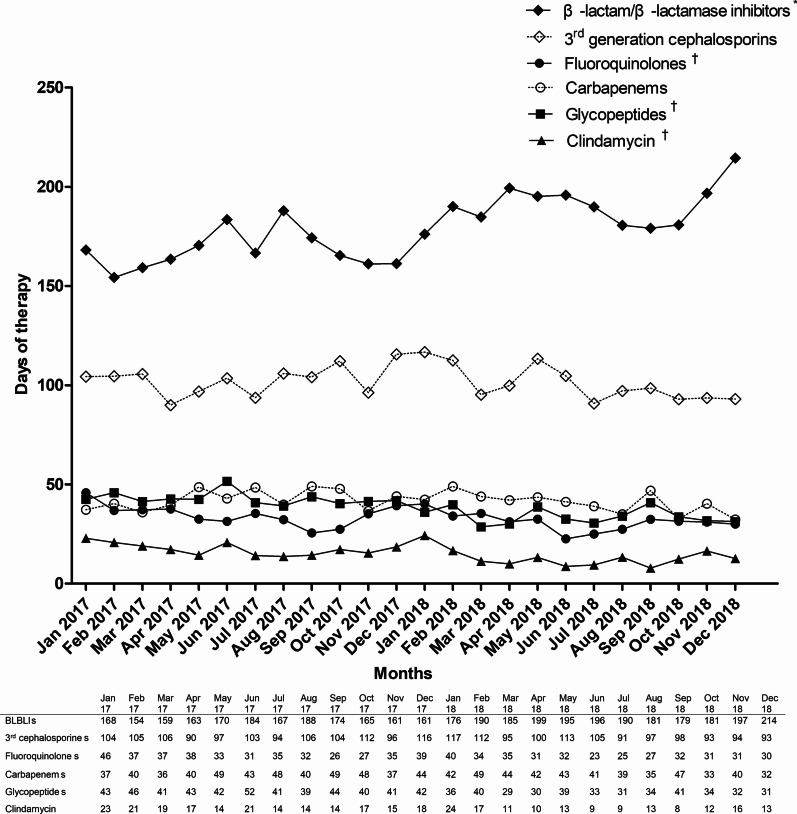
The trend of antibiotic consumption with days of therapy during the study period. The consumption of β-lactam/β-lactamase inhibitors significantly increased, while the consumption of fluoroquinolones, clindamycin, and glycopeptides significantly decreased. *Indicates the antibiotics whose use increased significantly and † indicates the antibiotics whose use decreased significantly. *BLBLIs* β-lactam/β-lactamase inhibitors, *3rd cephalosporins* third-generation cephalosporins

The consumption of all classes of intravenous antibiotics evaluated in this study, except BLBLIs, showed a decreasing trend in use; specifically, a significant decrease in fluoroquinolones, glycopeptides, and clindamycin. Fluoroquinolone use significantly decreased in terms of DOT (coefficient, − 0.39; *P* = 0.01), glycopeptide use significantly decreased in terms of DDD (coefficient, − 0.60; *P* < 0.001) and DOT (coefficient, − 0.61; *P* < 0.001), and clindamycin use significantly decreased in terms of DDD (coefficient, − 0.33; *P* = 0.003) and DOT(coefficient, − 0.39; *P* = 0.001). Conversely, consumption of BLBLI significantly increased in terms of DDD (from 145 to 187 per 1000 patient-days: coefficient, 1.30; *P* < 0.001) (Fig. 2) and DOT (from 168 to 214 per 1000 patient-days: coefficient, 1.58; *P* < 0.001) (Fig. 3). The total consumption of antibiotics showed a decreasing trend in terms of DDD and DOT, although this was not statistically significant.

### Correlation between the incidence of HO-CDI and antibiotic consumption

The consumption of most antibiotics, except BLBLIs and tigecycline, showed a negative correlation with the incidence of HO-CDI. The incidence of HO-CDI was significantly associated with the consumption of BLBLIs and glycopeptide in terms of DDD, and BLBLIs, third-generation cephalosporins, fourth-generation cephalosporins, and glycopeptides in terms of DOT (Table 1). Total antibiotic consumption was not significantly associated with the incidence of HO-CDI, either in terms of DDD or DOT. When we matched the 1-month interval, the consumption of glycopeptides and clindamycin showed a significant association with the incidence of HO-CDI in terms of DDD and DOT (Table 2).

**Table 1:** Correlation analysis between antibiotic consumption and the incidence of HO-CDI without a time interval

Class of antibiotics	DDD		DOT	
	Spearman ρ	*P* value	Spearman ρ	*P* value
Total	− 0.08	0.72	− 0.18	0.38
BLBLIs	0.50	0.01*	0.58	0.003*
Third-generation cephalosporins	− 0.33	0.11	− 0.42	0.04*
Fourth-generation cephalosporins	− 0.40	0.05	− 0.46	0.03*
Fluoroquinolones	− 0.14	0.52	− 0.38	0.07
Carbapenems	− 0.29	0.17	− 0.32	0.13
Glycopeptides	− 0.51	0.01*	− 0.54	0.01*
Tigecycline	0.12	0.57	0.10	0.64
Clindamycin	− 0.31	0.15	− 0.37	0.08

*BLBLIs* β-lactam/β-lactam inhibitors, *DDD* defined daily dose, *DOT* days of therapy, *HO-CDI* healthcare facility-onset *C. difficile* infection

*P < 0.05

**Table 2:** Correlation analysis between antibiotic consumption and the incidence of HO-CDI with 1-month interval matching

Class of antibiotics	DDD		DOT	
	Spearman ρ	*P* value	Spearman ρ	*P* value
Total	− 0.27	0.22	− 0.26	0.23
BLBLIs	0.36	0.10	0.40	0.06
Third-generation cephalosporins	− 0.35	0.10	− 0.28	0.20
Fourth-generation cephalosporins	− 0.26	0.22	− 0.25	0.25
Fluoroquinolones	0.03	0.89	− 0.12	0.58
Carbapenems	− 0.04	0.84	− 0.15	0.49
Glycopeptides	− 0.48	0.02*	− 0.45	0.03*
Tigecycline	0.17	0.43	0.18	0.40
Clindamycin	− 0.44	0.04*	− 0.49	0.02*

*BLBLI* β-lactam/β-lactam inhibitor, *DDD* defined daily dose, *DOT* days of therapy, *HO-CDI* healthcare facility-onset *C. difficile* infection

*P < 0.05

When the analysis was conducted for each antibiotic, the consumption of ampicillin/sulbactam (*P* = 0.003 [DDD] and *P* < 0.001 [DOT]), ceftriaxone (*P* = 0.02 [DOT]), cefotaxime (*P* = 0.04 [DOT]), cefepime (*P* = 0.03 [DOT]), meropenem (*P* = 0.045 [DOT]), vancomycin (*P* = 0.03 [DOT]), and teicoplanin (*P* = 0.04 [DDD] and *P* = 0.02 [DOT]) was significantly correlated with the incidence of HO-CDI. In the 1-month interval matching analysis, the incidence of HO-CDI was significantly correlated with the consumption of ampicillin/sulbactam (*P* = 0.02 [DOT]), vancomycin (*P* = 0.03 [DDD] and *P* = 0.047 [DOT]), and clindamycin (*P* = 0.04 [DDD] and *P* = 0.02 [DOT]) (Additional file 1: Tables S1 and S2). Among the antibiotics that showed a significant association with the incidence of HO-CDI, ampicillin/sulbactam and cefotaxime were the only antibiotics that positively correlated with the incidence of HO-CDI.

In univariate analysis, the consumption of BLBLIs and glycopeptides significantly correlated with the incidence of HO-CDI. These variables, and other variables with *P* < 0.1, were included in the regression model. Multivariate analysis included the consumption of BLBLIs, glycopeptides, and carbapenems for DDD, and the consumption of BLBLIs, third-generation cephalosporins, fluoroquinolones, carbapenems, and glycopeptides for DOT. The analysis showed that the consumption of BLBLIs (*P* = 0.002 [DDD] and *P* < 0.001 [DOT]) was independently associated with the incidence of HO-CDI (Table 3). The consumption of BLBLIs (*P* = 0.01 [DOT]) and glycopeptides (*P* = 0.03 [DDD] and *P* = 0.045 [DOT]) were also correlated with the incidence of HO-CDI in the sensitivity analysis, excluding the outlying data points (January 2017 and December 2018) (Additional file 1: Table S3). When the regression analysis was conducted with 1-month interval matching, the consumption of glycopeptides (*P* = 0.04 [DDD] and *P* = 0.02 [DOT]) was independently associated with the incidence of HO-CDI (Table 4).

**Table 3:** Regression analysis between antibiotic consumption and the incidence of HO-CDI

Risk factor	Univariate analysis^a^		Multivariate analysis^c^	
	Coefficient^b^	*P* value	Coefficient	*P* value
DDD				
BLBLIs	0.11	0.002	0.11	0.002
Carbapenems	− 0.18	0.10		
Glycopeptides	− 0.18	0.02		
DOT				
BLBLIs	0.11	< 0.001	0.11	< 0.001
Third-generation cephalosporins	− 0.11	0.07		
Fluoroquinolones	− 0.14	0.08		
Carbapenems	− 0.18	0.07		
Glycopeptides	− 0.24	0.01		

*BLBLIs* β-lactam/β-lactam inhibitors, *DDD* defined daily dose, *DOT* days of therapy, *HO-CDI* healthcare facility-onset *C. difficile* infection

^a^Variables with *P* < 0.1 in the univariate analysis were included in the multivariate analysis

^b^Changes in the incidence of HO-CDI according to antibiotic consumption

^c^R-squared in multivariate analysis: 0.36 [DDD] and 0.42 [DOT]

**Table 4:** Regression analysis between antibiotic consumption and the incidence of HO-CDI with 1-month interval matching

Risk factor	Univariate analysis^a^		Multivariate analysis^c^	
	Coefficient^b^	*P* value	Coefficient	*P* value
DDD				
BLBLIs	0.07	0.10		
Glycopeptides	− 0.16	0.04	− 0.16	0.04
DOT				
BLBLIs	0.07	0.045		
Glycopeptides	− 0.20	0.02	− 0.20	0.02
Clindamycin	− 0.21	0.07		

*BLBLIs* β-lactam/β-lactam inhibitors, *DDD* defined daily dose, *DOT* days of therapy, *HO-CDI* healthcare facility-onset *C. difficile* infection

^a^Variables with *P* < 0.1 in the univariate analysis were included in the multivariate analysis

^b^Changes in the incidence of HO-CDI according to antibiotic consumption

^c^R-squared in multivariate analysis: 0.19 [DDD] and 0.22 [DOT]

## Discussion

In this study, we evaluated changes in the incidence of HO-CDI and the consumption of antibiotics, and examined the association between the incidence of HO-CDI and the consumption of antibiotics. We found that the incidence of HO-CDI significantly increased during the study period, despite no increase in the total consumption of antibiotics in terms of DDD and DOT. The consumption of BLBLIs, third-generation cephalosporins, fourth-generation cephalosporins, and glycopeptides significantly correlated with the incidence of HO-CDI. Among these antibiotics, only the consumption of BLBLIs was significantly correlated with the incidence of HO-CDI, in the regression analysis.

The mean incidence of CDI was 9.3 episodes per 10,000 patient-days in our study. Previous studies reported various incidences of CDI according to the instrument, with a range of 2.8 to 9.3 per 10,000 patient-days [6]. In Korea, the mean incidence of HO-CDI was reported to be 7.16 episodes per 10,000 patient-days, in one study [21]. Our results were in the upper range of those reported in previous studies. The incidence of HO-CDI increased during our study period, despite no change in the total consumption of intravenous antibiotics. During the study period, the total number of laboratory tests to detect CDI increased from 3287 in 2017 to 4126 in 2018. The wide use of highly sensitive tests, such as a NAAT and glutamate dehydrogenase assay, may enable the detection of HO-CDI cases more precisely.

The pattern of antibiotic consumption changed during the study period. The major findings were a decrease in the consumption of glycopeptides, fluoroquinolones, and clindamycin, and an increase in the consumption of BLBLIs. These findings suggest that the antibiotic stewardship program works appropriately. To reduce multidrug-resistant pathogens, such as methicillin-resistant *Staphylococcus aureus*, extended-spectrum β-lactamase producing gram-negative bacilli, carbapenem-resistant gram-negative bacilli, and vancomycin-resistant enterococci, we restricted the inappropriate use of broad-spectrum antibiotics, such as carbapenems, fluoroquinolones, and glycopeptides. This intervention may influence the “balloon effect” in which physicians use BLBLIs more frequently. Therefore, we could not reduce the total consumption of antibiotics significantly during the study period. The “balloon effect” may reduce the effectiveness of the antibiotic stewardship program.

The consumption of BLBLIs and the incidence of HO-CDI showed a significant relationship. Similarly, Vernaz et al. [22] reported that amoxicillin/clavulanate consumption was correlated with the incidence of HO-CDI. However, some studies reported that the use of BLBLIs was associated with a lower incidence of HO-CDI than the use of high-risk antibiotics, such as third-generation cephalosporins and fluoroquinolones [23–28]. In our study, the risk and relative risk of HO-CDI with specific classes of antibiotics were not evaluated. Therefore, our results did not suggest that BLBLIs were associated with a higher risk of HO-CDI than other classes of antibiotics. Restricting the use of specific classes of antibiotics, such as third-generation cephalosporins and fluoroquinolones, may have a limited effect on the incidence of HO-CDI.

The consumption of ampicillin/sulbactam and amoxicillin/clavulanate, but not piperacillin/tazobactam, were significantly correlated with the incidence of HO-CDI. In previous studies, use of piperacillin/tazobactam was associated with a lower risk of *C. difficile* colonization and CDI, compared with the consumption of third-generation cephalosporins [23–27]. In studies that reported on the antibiotic susceptibility of *C. difficile* strains in Korea, almost all isolated *C. difficile* strains were susceptible to piperacillin/tazobactam, while approximately half of *C. difficile* isolates were resistant to ampicillin [29, 30]. The antibiotic susceptibility of *C. difficile* may influence the occurrence of HO-CDI. Further studies are needed to establish the impact of antibiotic susceptibility on the incidence of HO-CDI.

This study had some limitations. First, we only evaluated the consumption of antibiotics and the incidence of HO-CDI. Because of the ecological nature of this study, individual risk factors, such as age, underlying diseases, and disease severity, and the impact of changes in infection control measures could not be evaluated. Only a population-level correlation between the consumption of antibiotics and the incidence of HO-CDI could be evaluated. Second, the study period was relatively short. In our hospital, a NAAT and glutamate dehydrogenase assay were introduced in September 2016. Before then, toxin assays using enzyme immunoassays and toxigenic cultures for *C. difficile* were used to detect CDI. To minimize the impact of the changes in diagnostic tests, we used data after September 2016. Lastly, we did not evaluate the strain and antibiotic susceptibility of *C. difficile* detected in our study. Therefore, evaluation of the effect of the strain and antibiotic susceptibility on the incidence of HO-CDI was limited. Despite these limitations, our study showed a significant correlation between the consumption of BLBLIs and the incidence of HO-CDI in terms of DDD and DOT.

## Conclusions

During the study period, the incidence of HO-CDI and the consumption of BLBLIs significantly increased, while the total antibiotic consumption did not. The consumption of BLBLIs was associated with the incidence of HO-CDI. Reduced consumption of specific antibiotics only may be insufficient to reduce the incidence of HO-CDI.

## Supplementary Information

**Additional File 1: Table 1**. Correlation analysis between antibiotic consumption and the incidence of HO-CDI without a time interval.

## Data Availability

All data generated or analyzed during this study are included in this published article [and its supplementary information files).

## Abbreviations

BLBLIs: β-Lactam/β-lactamase inhibitors
CDI: *Clostridioides difficile* Infection
DDD: Defined daily dose
DOT: Days of therapy
HO-CDI: Healthcare facility-onset *Clostridioides difficile* infection
NAAT: Nucleic acid amplification test
