# Supplementary material for: Correlation between antibiotic consumption and the incidence of healthcare facility-onset Clostridioides difficile infection: a retrospective chart review and analysis
Source: Antimicrob Resist Infect Control. 2021 Aug 6;10:117. doi: 10.1186/s13756-021-00986-9 (PMC8348999; doi:10.1186/s13756-021-00986-9)
Supplement: Supplementary file 1 — Additional File 1: Table 1. Correlation analysis between antibiotic consumption and the incidence of HO-CDI without a time interval. [file 13756_2021_986_MOESM1_ESM.docx]

**Additional Table 1.** Correlation analysis between antibiotic consumption and the incidence of HO-CDI without a time interval

|  | **DDD** |  | **DOT** |  |
| --- | --- | --- | --- | --- |
| **Class of antibiotic** | **Spearman ρ** | ***P*-value** | **Spearman ρ** | ***P*-value** |
| BLBLIs |  |  |  |  |
| Ampicillin/sulbactam | 0.58 | 0.003* | 0.65 | < 0.001* |
| Amoxicillin/clavulanate | –0.11 | 0.61 | –0.18 | 0.39 |
| Piperacillin/tazobactam | 0.31 | 0.15 | 0.29 | 0.17 |
| Third-generation cephalosporins |  |  |  |  |
| Ceftriaxone | −0.27 | 0.20 | −0.47 | 0.02* |
| Cefotaxime | 0.24 | 0.27 | 0.42 | 0.04* |
| Ceftazidime | −0.26 | 0.24 | −0.2 | 0.36 |
| Cefpodoxime | –0.13 | 0.54 | –0.07 | 0.73 |
| Cefixime | 0.00 | > 0.99 | 0.04 | 0.86 |
| Cefditoren | –0.05 | 0.81 | –0.06 | 0.77 |
| Cefdinir | –0.13 | 0.55 | –0.13 | 0.54 |
| Cefepime | −0.40 | 0.05 | −0.46 | 0.03* |
| Fluoroquinolones |  |  |  |  |
| Ciprofloxacin | –0.25 | 0.24 | –0.35 | 0.09 |
| Levofloxacin | –0.01 | 0.96 | –0.10 | 0.65 |
| Moxifloxacin | –0.06 | 0.77 | –0.06 | 0.78 |
| Carbapenems |  |  |  |  |
| Ertapenem | −0.05 | 0.83 | −0.01 | 0.96 |
| Imipenem | 0.29 | 0.17 | 0.36 | 0.08 |
| Meropenem | −0.30 | 0.15 | −0.41 | 0.045* |
| Glycopeptides |  |  |  |  |
| Vancomycin | −0.38 | 0.07 | −0.45 | 0.03* |
| Teicoplanin | −0.43 | 0.04^*^ | −0.46 | 0.02* |
| Tigecycline | 0.12 | 0.57 | 0.10 | 0.64 |
| Clindamycin | −0.31 | 0.15 | −0.37 | 0.08 |

* P < 0.05

BLBLIs, β-lactam/β-lactam inhibitors; DDD, defined daily dose; DOT, days of therapy; HO-CDI, healthcare facility-onset *C. difficile* infection.

**Additional Table 2.** Correlation analysis between antibiotic consumption and the incidence of HO-CDI with 1-month interval matching

| **Class of antibiotics** | **DDD** |  | **DOT** |  |
| --- | --- | --- | --- | --- |
|  | **Spearman ρ** | ***P*-value** | **Spearman ρ** | ***P*-value** |
| BLBLIs |  |  |  |  |
| Ampicillin/sulbactam | 0.38 | 0.07 | 0.48 | 0.02* |
| Amoxicillin/clavulanate | 0.24 | 0.28 | 0.03 | 0.89 |
| Piperacillin/tazobactam | −0.03 | 0.90 | −0.05 | 0.82 |
| Third-generation cephalosporins |  | | | |
| Ceftriaxone | −0.34 | 0.12 | −0.32 | 0.14 |
| Cefotaxime | −0.04 | 0.87 | 0.11 | 0.61 |
| Ceftazidime | 0.11 | 0.62 | 0.11 | 0.63 |
| Cefpodoxime | 0.02 | 0.93 | 0.04 | 0.84 |
| Cefixime | 0.21 | 0.34 | 0.13 | 0.55 |
| Cefditoren | –0.29 | 0.18 | –0.29 | 0.18 |
| Cefdinir | –0.29 | 0.19 | –0.34 | 0.12 |
| Cefepime | −0.26 | 0.22 | −0.25 | 0.25 |
| Fluoroquinolones |  |  |  |  |
| Ciprofloxacin | 0.02 | 0.93 | −0.08 | 0.72 |
| Levofloxacin | 0.12 | 0.60 | 0.11 | 0.61 |
| Moxifloxacin | –0.15 | –0.49 | –0.19 | 0.39 |
| Carbapenems |  |  |  |  |
| Ertapenem | 0.02 | 0.92 | −0.03 | 0.91 |
| Imipenem | 0.06 | 0.80 | 0.12 | 0.57 |
| Meropenem | −0.16 | 0.48 | −0.29 | 0.17 |
| Glycopeptides |  |  |  |  |
| Vancomycin | −0.46 | 0.03* | −0.42 | 0.047* |
| Teicoplanin | −0.33 | 0.13 | −0.39 | 0.07 |
| Tigecycline | 0.17 | 0.43 | 0.18 | 0.40 |
| Clindamycin | −0.44 | 0.04* | −0.49 | 0.02* |

* *P* < 0.05

BLBLIs, β-lactam/β-lactam inhibitors; DDD, defined daily dose; DOT, days of therapy; HO-CDI, healthcare facility-onset *C. difficile* infection.

**Additional Table 3.** Univariate analysis between antibiotic consumption and the incidence of HO-CDI (February 2017 to November 2018)

| **Class of antibiotics** | **DDD** |  | | **DOT** |  | |
| --- | --- | --- | --- | --- | --- | --- |
|  | **Coefficient^a^** | | ***P*-value** | **Coefficient** | | ***P-*value** |
| BLBLIs | 0.07 | | 0.06 | –0.02 | | 0.52 |
| Third-generation cephalosporins | –0.08 | | 0.15 | 0.07 | | 0.02 |
| Fourth-generation cephalosporins | –0.07 | | 0.30 | –0.07 | | 0.17 |
| Fluoroquinolones | –0.02 | | 0.87 | –0.09 | | 0.22 |
| Carbapenems | –0.14 | | 0.14 | –0.12 | | 0.17 |
| Glycopeptides | –0.13 | | 0.04 | –0.16 | | 0.02 |
| Tigecycline | 0.14 | | 0.41 | 0.17 | | 0.36 |
| Clindamycin | –0.05 | | 0.67 | –0.06 | | 0.55 |

^a^ Changes in the incidence of HO-CDI according to antibiotic consumption

BLBLIs, β-lactam/β-lactam inhibitors; DDD, defined daily dose; DOT, days of therapy; HO-CDI, healthcare facility-onset *C. difficile* infection.
